# Association between gout and kidney stone: results from mendelian randomization and the NHANES study

**DOI:** 10.3389/fgene.2024.1417663

**Published:** 2024-10-24

**Authors:** Shengkai Jin, Haochen Geng, Yichen Lu, Yuhua Zhou, Jing Lv, Chaowei Fu, Yuwei Zhang, Menglu Li, Ninghan Feng

**Affiliations:** ^1^ Wuxi School of Medicine, Jiangnan University, Wuxi, China; ^2^ Department of Urology, Jiangnan University Medical Center, Wuxi No. 2 People’s Hospital, Wuxi, China; ^3^ School of Medicine, Nanjing Medical University, Nanjing, China; ^4^ School of Medicine, Nantong University, Nantong, China

**Keywords:** gout, kidney stone, national health and nutrition examination survey, cross-sectional research, mendelian randomization

## Abstract

**Background:**

Kidney stones are a common urologic disease with an increasing incidence year by year, and there are similar influences between gout status and kidney stone incidence. Therefore the contribution of gout status to the incidence of kidney stones is unclear. The aim of this study was to investigate the relationship between gout status and kidney stones and to further explore the causal relationship by Mendelian randomization (MR) analysis.

**Method:**

An epidemiologic study of 49,693 participants in the 2009–2018 National Health and Nutrition Examination Survey (NHANES) was conducted to examine the association between the two. The causal relationship between gout status and kidney stones was assessed by Mendelian randomization analysis of data from the GWAS database.

**Result:**

A total of 28,742 participants were included in the NHANES analysis. We found that gout status was associated with an increased risk of kidney stones [odds ratio (OR) = 1.45 (95%CI, 1.243–1.692); *p* < 0.001]. In the MR analysis, we found a causal relationship between gout status and the risk of developing kidney stones (OR = 1.047, 95%CI, 1.011–1.085, *p* = 0.009).

**Conclusion:**

There may be an association between gout status and kidney stone risk. This finding requires further large-sample studies and adequate follow-up.

## Introduction

Kidney stones are a kidney disease with a global and increasing incidence (Peerapen and Thongboonkerd, 2023). It has been reported that the incidence of kidney stones in the United States has increased from 3.2% to 9.6% from 1980 to the present (Di X et al., 2023). Kidney stones are a huge economic burden, costing approximately billions of dollars annually (Litwin et al., 2005). Symptomatic kidney stones are more prevalent in men compared with women (Kittanamongkolchai et al., 2018). According to epidemiologic surveys, the highest incidence of kidney stones was found in men after the age of 60, and in women at the age of 50 (Lieske et al., 2006). This suggests a potential correlation between the causes of kidney stones and gender and age. The occurrence of kidney stones not only reduces the quality of life of the patient but can also be life-threatening (Bryant et al., 2012; Whitehurst et al., 2019). Although there are many treatment strategies available for kidney stones, it is more important to prevent their occurrence.

It is well known that there is a significant correlation between gout and kidney stones (Kim et al., 2017). Gout is a chronic inflammatory disease caused by excessive deposition of uric acid crystals in joints and non-joint structures (Dalbeth et al., 2021). Meanwhile, gout is the most common inflammatory arthritis (Keller and Mandell, 2021). And the prevalence and disability of gout worldwide is increasing yearly (McCormick et al., 2022). There is a correlation between gout and the development of several kidney diseases, and a correlation has been reported between elevated urate levels and the development of kidney stones (Ramos and Goldfarb, 2022). This suggests that gout may play a crucial role in the prevention of kidney stones.

Mendelian randomization (MR) is an emerging and valuable approach to causality research. It uses single nucleotide polymorphisms (SNPs) as instrumental variables to the causal relationship between inflammatory exposures (e.g., gout) and outcomes (e.g., kidney stones). Because gene alleles are randomly assigned during meiosis independent of environmental factors, genetic associations observed from MR analyses are unlikely to be affected by confounding bias and risk of reverse causation (Li et al., 2023). Because the method is effective in avoiding confounding factors from interfering with the analyzed results, MR is widely used for causal hypothesis studies in epidemiology.

In this study, we investigate the causal relationship between gout and kidney stones by combining the National Health and Nutrition Examination Survey (NHANES) and MR analysis.

## Materials and methods

### Study population in NHANES

**FIGURE 1:**
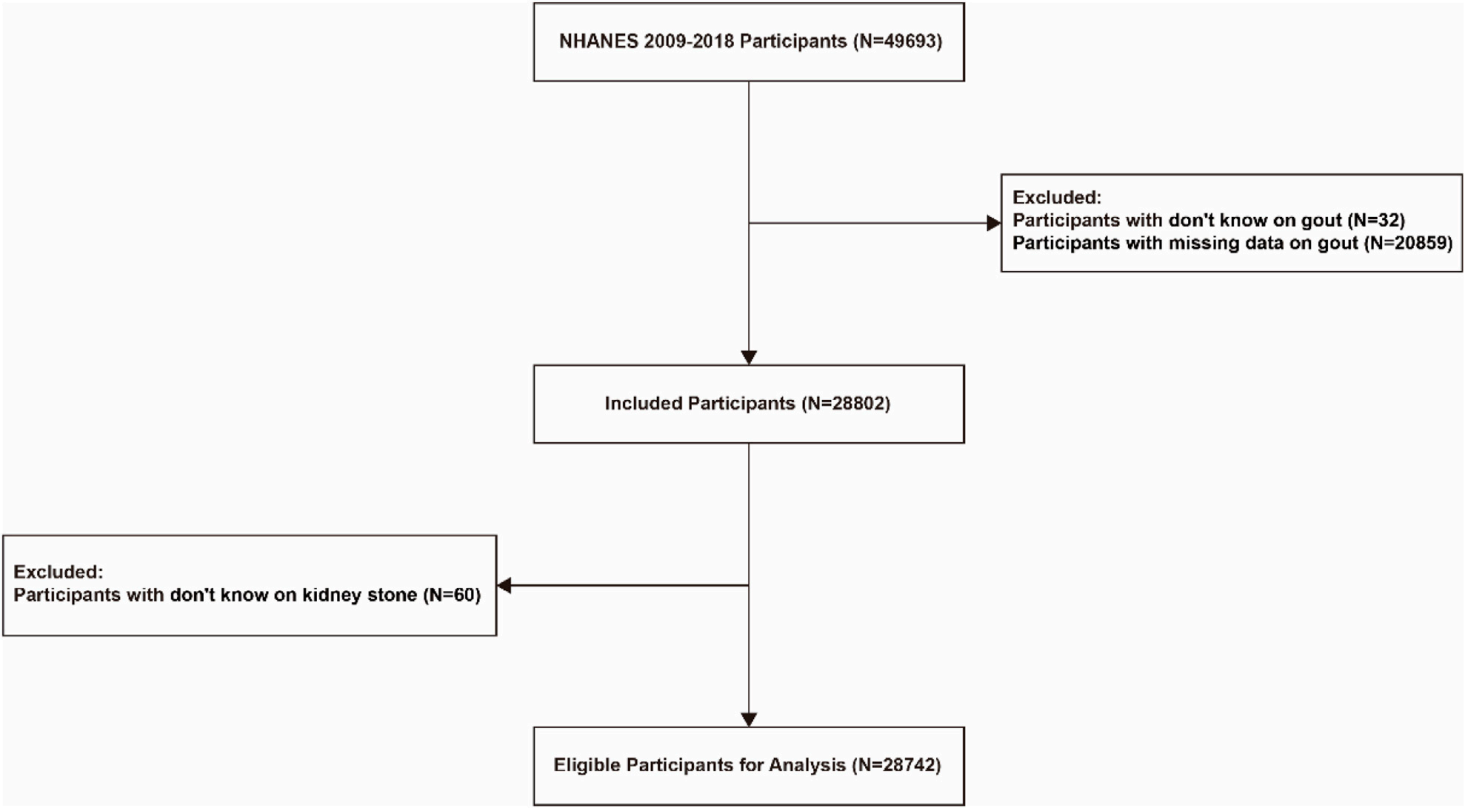
Flow chart for inclusion of participants based on inclusion and exclusion criteria. Schematic diagram showing study participants included for the present analysis from the 2009 to 2018 NHANES. A total of 28,742 participants were included. NHANES, National Health and Nutrition Examination Survey.

The National Health and Nutrition Examination Survey (NHANES) is a 2-year cross-sectional study designed to investigate the health status of the U.S. population. Data on sociodemographic characteristics, diseases, and related aspects are obtained through questionnaires and laboratory data. Gout was labeled in the database as “Doctor ever told you that you had gout?”. Kidney stones were labeled as “Ever had kidney stones? “To make our findings more accurate, we selected data from 2009–2018 (N = 49,693) for analysis. In this study, we excluded patients with “don't know” and “missing data” from the gout data, and then excluded patients with “don't know” from the kidney stones, and finally included the remaining 28,742 patients in our study (Figure 1).

### Covariates used in NHANES

To control for potential confounders, we chose the following demographic characteristics: sex, age, race, education level, BMI (body mass index), smoking, alcohol consumption, sleep disorders, uric acid levels, and water consumption. These variables were chosen because of their possible association with outcomes. Participants were categorized as smokers and nonsmokers based on whether they had smoked fewer than 100 cigarettes in their lifetime. Participants were categorized as drinkers, nondrinkers, refused to answer, and don't know based on whether they had more than 12 drinks in their lifetime. Participants were categorized as sleep-disordered and non-sleep-disordered based on whether they had a sleep disorder. Uric acid levels were categorized as < 4 mg/dL, 4–7 mg/dL, and > 7 mg/dL based on circulating uric acid levels.

### Data sources for exposure and outcomes

Genetic variants strongly associated with gout were obtained from the IEU OPEN GWAS, and we chose the gout number “ukb-a-107”. A total of 337,159 investigators (332,352 controls and 4,807 cases) were included in this data. Genetic variants associated with kidney stones were obtained from UK biobank, number “ukb-a-72”, which contained 337,159 investigators (334,465 controls and 2,694 cases).

### Selection of genetic instruments

Instrumental variables (single nucleotide polymorphisms, SNPs) were screened by the following criteria: 1) significant gene-wide correlation (*p* < 5 × 10^−8^); 2) pruned by linkage disequilibrium (r^2^ < 0.001 and within 10,000 kb from the index variant).

### Statistical analysis

In our observational study, we selected appropriate data in the NHANES database and used multivariate logistic regression to assess the association between gout status and kidney stone risk. In addition, we used the sample weights provided by NHANES in our observational study.

The validity of the results of the MR analysis depends on the key assumption of no pleiotropy (Lawlor et al., 2008). First, we applied the MR-Egger method, which is based on the Instrument Strength Independent of Direct Effect assumption. In this method, the effect of SNPs on gout is charted against the effect of SNPs on kidney stone, and an intercept dissimilar from that of the source is considered as proof of pleiotropic effects (Bowden et al., 2015; Bowden et al., 2015). This method yields bias-free estimations even if all the selected SNPs are unfounded. Furthermore, we used three more MR models that are more robust under horizontal pleiotropy conditions: simple mode, weighted mode, weighted median (Burgess et al., 2016; Li et al., 2023; Lin et al., 2023). We used Cochrane’s Q test to test for possible SNP heterogeneity. We also performed omission analysis and MR-Egger intercept tests to assess significant estimates of horizontal pleiotropy. MR analyses were performed using the R package “TwoSampleMR”, recognizing outliers with the “MR-PRESSO” package (Burgess et al., 2016; Li et al., 2023; Lin et al., 2023). All results are reported as odd ratios, with 95% confdence intervals (CI). All statistical analysis and data visualization were performed in R software 4.2.0 (https://www.r-project.org/).

## Result

### Observations on gout and kidney stones in NHANES

By selection, 28,742 participants were included in our study (Figure 1). Table 1 shows the Baseline characteristics of participants by gout in the NHANES 2009–2018. The age range of the participants was 20–80 years old, with 51.6% females and 48.4% males. Gout was diagnosed by doctors in 3.2% of the men and 1.5% of the women. The occurrence of gout was associated with age (64 vs. 49 years; *p* < 0.001) and was more likely to be associated with males (67.6% vs. 32.4%; *p* < 0.001) and non-Hispanic whites (46.4% vs. 38.8%; *p* < 0.001), and with a BMI > 30 kg/m^2^ (53.8% vs. 38.0%; *p* < 0.001). In addition, the prevalence of kidney stones (16.6% vs. 9.0%; *p* = 0.004) and whether sleep difficulties (39% vs. 25.2%; *p* < 0.001) were significantly higher in gout patients than in non-gout patients among all participants. Gout patients had higher rates of uric acid > 7 mg/dL (*p* < 0.001), smoking (*p* < 0.001), alcohol consumption (*p* < 0.001) and sleep difficulties (*p* < 0.001) compared to non-gout patients. Gout patients had higher rates of uric acid > 7 mg/dL (*p* < 0.001), smoking (*p* < 0.001), alcohol consumption (*p* < 0.001), and sleep difficulties (*p* < 0.001) compared to healthy individuals. Among patients with gout, 16.6% had a history of kidney stones. In contrast, the prevalence of gout among patients reporting kidney stones was 8.4%.

**TABLE 1:** Baseline characteristics of participants by gout in the NHANES 2009–2018.

	Gout	Non-gout	*P*-Value
Total number of participants	1356	27,386	
Kidney stones (%)			0.004
Yes	225 (16.6)	2466 (9.0)	
No	1131 (83.4)	24,920 (91.0)	
Age, median (Years)	64	49	<0.001
Gender (%)			<0.001
Male	917 (67.6)	12,983 (47.4)	
Female	439 (32.4)	14,403 (52.6)	
Race/ethnicity (%)			<0.001
Mexican American	94 (6.9)	4068 (14.9)	
Other Hispanic	77 (5.7)	2906 (10.6)	
Non-Hispanic White	629 (46.4)	10,627 (38.8)	
Non-Hispanic Black	373 (27.5)	5864 (21.4)	
Other raceincluding multiracial	183 (13.5)	3921 (14.3)	
Education level (%)			0.738
Less than 9th grade	149 (11.0)	2773 (10.2)	
9–11th grade	204 (15.0)	3674 (13.5)	
High school graduate/GED or equivalent	326 (24.0)	6111 (22.3)	
Some college or AA degree	401 (29.6)	8220 (30.0)	
College graduate or above	276 (20.4)	6567 (24.0)	
BMI category (%)			<0.001
<25 kg/m^2^	213 (15.7)	8022 (29.3)	
25–30 kg/m^2^	413 (30.5)	8967 (32.7)	
>30 kg/m^2^	730 (53.8)	10,397 (38.0)	
Smoked at least 100 cigarettes in life (%)			<0.001
Yes	786 (58.0)	11,656 (42.6)	
No	570 (42.0)	15,713 (57.4)	
Had at least 12 alcohol drinks/lifetime? (%)			<0.001
Yes	887 (65.4)	18,126 (66.2)	
No	422 (31.1)	8977 (32.8)	
Refused	45 (3.3)	202 (0.7)	
Don’t know	2 (0.2)	81 (0.3)	
Whether you have trouble sleeping? (%)			<0.001
Yes	529 (39.0)	6892 (25.2)	
No	827 (61.0)	20,482 (74.8)	
Uric acid (mg/dL)			<0.001
<4 mg/dL	92 (6.8)	4235 (15.5)	
4–7 mg/dL	761 (56.1)	19,805 (72.3)	
>7 mg/dL	503 (37.1)	3346 (12.2)	
Daily water intake, median(g)	2871.74	2893.77	0.034

Data are presented as n (%). N is the total number of patients with available data. *p*-value < 0.05 was considered statistically significant. BMI, body mass index.

In the age-adjusted model, kidney stones were significantly associated with gout in the study population [odds ratio (OR) = 1.45; 95%CI, 1.243–1.692] (Table 2). In the multivariate model, we found that gout was significantly and positively associated with kidney stones (OR = 1.284; 95%CI, 1.084–1.522) (Table 3). However, in the multivariate model, the result for educational level was not statistically significant (*p* = 0.738 > 0.05), so the difference in educational level was not significant among gout patients.

**TABLE 2:** Relationship between age, sex, kidney stones and gout.

	Beta value	Or (95% CI)	*p*-Value
Gender	−0.841	0.431 (0.383–0.485)	<0.001
Age in years at screening	0.053	1.054 (1.05–1.058)	<0.001
Had kidney stone	0.372	1.45 (1.243–1.692)	<0.001

**TABLE 3:** The relationship between multivariate and gout.

	Beta value	Or (95% CI)	*p*-Value
Gender	−0.722	0.486 (0.424–0.557)	<0.001
Age in years at screening	0.052	1.053 (1.049–1.058)	<0.001
Race/Hispanic origin	0.273	1.313 (1.235–1.397)	<0.001
Education level - Adults 20+	−0.008	0.992 (0.944–1.042)	0.738
Smoked at least 100 cigarettes in life	−0.247	0.781 (0.692–0.883)	<0.001
Ever told doctor had trouble sleeping?	−0.511	0.6 (0.528–0.681)	<0.001
Ever had a drink of any kind of alcohol	0.154	1.167 (1.102–1.236)	<0.001
Uric Acid Group	0.802	2.229 (1.977–2.513)	<0.001
BMI Group	0.451	1.57 (1.44–1.712)	<0.001
Moisture (gm)	0	1 (1–1)	0.034
Had kidney stones	0.25	1.284 (1.084–1.522)	0.004

### Causal association between gout and kidney stones in MR

**FIGURE 2:**
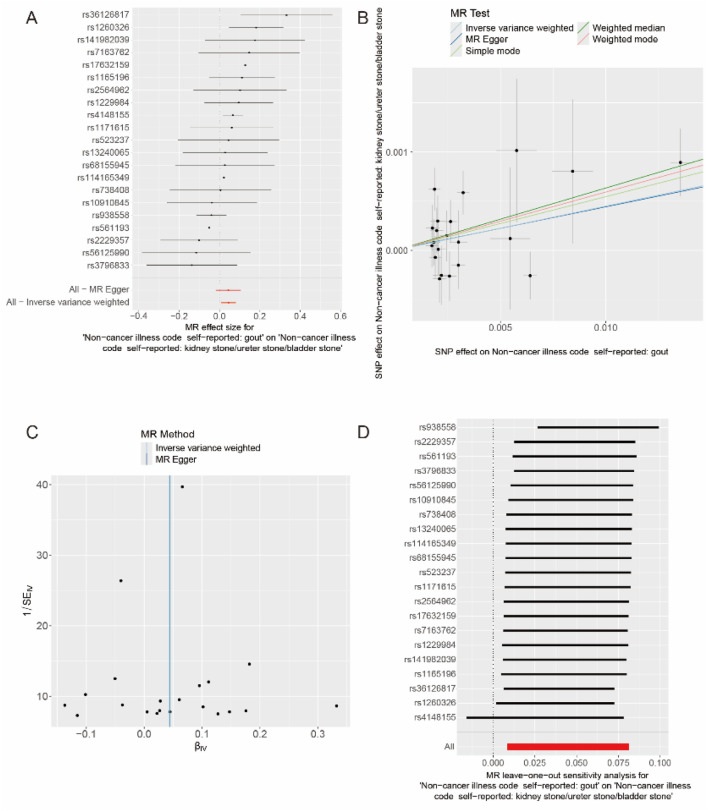
Causal relationship between gout and kidney stones. **(A)** Forest map of gout and kidney stones. **(B)** Scatter plot of gout and kidney stones. **(C)** Funnel plot of gout and kidney stones. **(D)** Results of the “leave-one-out” method in gout and kidney stones.

After data merging preprocessing, we selected a total of 22 SNPs for MR analysis (Figure 2A). We performed 5 MR analyses, in which the results of IVW, Weighted media, and Weighted mode were significant (*p* < 0.05) (Table 4). IVW showed that there was a causal relationship between gout and kidney stones (OR = 1.047; 95%CI,1.011–1.085, *p* = 0.009). Weighted media (OR = 1.065; 95%CI,1.016–1.116, *p* = 0.007) and Weighted mode (OR = 1.067; 95%CI, 1.010–1.128, *p* = 0.030) as sensitivity analyses similarly confirmed this. In contrast, MR Egger (OR = 1.044; 95%CI, 0.983–1.110, *p* = 0.174) and Simple mode (OR = 1.072, 95%CI, 0.968–1.187, *p* = 0.190) turned out to be non-significant (*p* > 0.05). However, the IVW, which provided a precise causal estimate, had a *p* = 0.009 < 0.05, and the beta values of all five tests were >0 in the same direction (Figure 2B), i.e., individuals who developed gout were at increased risk of kidney stones, which suggests that there is indeed a causal relationship between gout and kidney stones. In order to exclude the heterogeneity of SNPs affecting the results causing bias, we subsequently performed a heterogeneity analysis (Table 5), using Cochran’s Q test to assess the degree of heterogeneity of individual effects on the results, in which the Q test *P* for IVW (Q = 27, *p* = 0.145) as well as for MR-Egger (Q = 27, *p* = 0.113) values were > 0.05 suggesting that there is no heterogeneity,the effect of gout on kidney stones does not vary from individual to individual. Funnel plot results show that SNPs are symmetric, suggesting stable results (Figure 2C). In the test of multiplicity (Table 6), we verified the MR-Egger (except = 1.26e-05, *p* = 0.910), *p* = 0.910 > 0.05 proved the weak possibility of horizontal multiplicity, i.e., SNP directly affects the risk of renal stone but not gout, which can be ignored. After passing the tests for heterogeneity and pleiotropy, we performed leave-one-out analysis (Figure 2D) and observed that the gradual elimination of each SNP had little effect on the final results, with little change in the overall error line, establishing that it was not a single SNP that was responsible for the association between gout and kidney stone risk.

**TABLE 4:** Mendelian randomization estimates for the association between gout and kidney stone risk.

Analytical methods	OR	95%CI		*p*-Value
		Lower limit	Upper limit	
IVW	1.047	1.011	1.085	0.009
Weighted media	1.065	1.016	1.116	0.007
Weighted mode	1.067	1.010	1,128	0.030
Simple mode	1.072	0.968	1.187	0.190
MR Egger	1.044	0.983	1.110	0.174

OR, odds ratio; CI, confidence interval; IVW, inverse variance weighted.

**TABLE 5:** Heterogeneity test results of gout and kidney stone risk.

Method	Q	Q_df	Q_pval
MR Egger	27.81108	20	0.11394
IVW	27.82900	21	0.14506

**TABLE 6:** Pleiotropic results of gout and kidney stone risk.

Egger_intercept	se	*p*-Value
1.261272e^-^05	0.00011	0.91075

## Discussion

In this study, we conducted the first large-scale investigation of the association between gout status and kidney stone risk based on the NHANES database and MR analysis. We investigated the relationship between gout status and kidney stone risk and the potential causal relationship between them. Our results suggest that gout status is positively associated with kidney stone risk and that there is a causal relationship between them.

Previously, some researchers have explored the causal relationship between serum uric acid and the risk of urolithiasis. Ravi K. Narang et al. conducted a large-scale MR analysis comprising 359,827 participants, and the results showed that the existence of a causal relationship between serum uric acid and the risk of urolithiasis was not supported (Narang et al., 2021). However, in our cross-sectional study (2009–2018) based on the NHANES database, we found a significant positive association between gout status and kidney stone risk (*p* = 0.004). A significant correlation between patients with urinary tract stones and gout was found based on a previous case-finding study, which is consistent with our findings (Marchini et al., 2013).

The diagnosis of gout relies heavily on circulating levels of uric acid, and the most common components of urinary stones are: calcium oxalate, carbapatite, urate, struvite, and brushite (Ludwig and Matlaga, 2018; Ye et al., 2020). In the present study, our large investigative study in NHANES and MR analyses went on to explore whether gout status is a risk factor for kidney stones. Although the correlation between gouty status and kidney stones was demonstrated in our study, correlation studies between the two are scarce and further research is needed. In our MR analysis, more than half of the analytical methods supported a causal relationship between gout status and high risk of kidney stones. In our study, the result of MR-Egger (OR = 1.044, 95%CI, 0.983–1.110, *p* = 0.174) was not significant (*p* > 0.05), which suggests that horizontal pleiotropy does not exist in this study (Luo et al., 2021). Although the result of Simple mode (OR 1.072, 95%CI, 0.968–1.187, *p* = 0.190) was also not significant (*p* > 0.05), the results of our study were based on IVW(Yang et al., 2023).

Although the definitive mechanism between gout and kidney stones is not known, we discuss several possible hypotheses. First, gout patients have circulating levels of uric acid that are significantly higher than those of healthy individuals, and acidification of the urine due to high uric acid levels favors the formation of kidney stones (Ferraro et al., 2020). Second, the disease state of gout patients is accompanied by an unpleasant state, and the persistence or frequency of the unpleasant state leads to the development of kidney stones (Wang et al., 2022). A previous study conducted a meta-analysis of the correlation between gout and depression and anxiety, which showed a positive correlation between gout and mental health disorders (Howren et al., 2021). Meanwhile, a study reported a positive correlation between the degree of depression and the risk of kidney stones (Wang et al., 2022). However, the clear mechanism of the related studies still needs further research.

The main strength of this study was the use of observational studies from 2009–2018 in the NHANES database combined with MR analysis. The combined collation of many factors and the large sample size enabled us to adjust the analysis of confounders in the regression model in a timely manner and with sufficient computational power to analyze the causal relationship between gout and kidney stones. Observational studies alone can only illustrate the correlation between observed factors, whereas MR analysis further analyzes the causal relationship between observed factors on the basis of correlation analysis, and MR analysis can avoid the bias of results caused by confounding factors (Zuber et al., 2022). However, there are some limitations of our study. First, gout-related data in the NHANWS database were incomplete in recent years due to the impact of neocoronary pneumonia, which affected the further refinement of the results analyzed. Second, the study population was limited to the Americas and Europe, which may have had an impact on the generalizability of the results.

## Conclusion

In conclusion, our study demonstrated a strong positive correlation between gout status and the risk of developing kidney stones. And our MR analysis results also proved the causal relationship between gout status and kidney stones. However, our findings still need to be further validated and their underlying mechanisms need to be further investigated.

## Data Availability

The data that support the findings of this study are openly available at https://wwwn.cdc.gov/nchs/nhanes/Default.aspx and http://gwas.mrcieu.ac.uk
